# A Predictive Model for Large-for-Gestational-Age Infants among Korean Women with Gestational Diabetes Mellitus Using Maternal Characteristics and Fetal Biometric Parameters

**DOI:** 10.3390/jcm11174951

**Published:** 2022-08-23

**Authors:** Hee-Sun Kim, Soo-Young Oh, Geum Joon Cho, Suk-Joo Choi, Soon Cheol Hong, Ja-Young Kwon, Han Sung Kwon

**Affiliations:** 1 Division of Maternal-Fetal Medicine, Department of Obstetrics and Gynecology, Dongguk University Ilsan Hospital, Goyang 10326, Korea; 2 Division of Maternal-Fetal Medicine, Department of Obstetrics and Gynecology, Samsung Medical Center, Sungkyunkwan University School of Medicine, Seoul 06351, Korea; 3 Division of Maternal-Fetal Medicine, Department of Obstetrics and Gynecology, Korea University College of Medicine, Seoul 02841, Korea; 4 Division of Maternal-Fetal Medicine, Department of Obstetrics and Gynecology, Yonsei University College of Medicine, Yonsei University Health System, Seoul 03722, Korea; 5 Division of Maternal-Fetal Medicine, Department of Obstetrics and Gynecology, Konkuk University School of Medicine 120-1, Neungdongno, Gwangjin-gu, Seoul 05030, Korea

**Keywords:** ultrasound, gestational diabetes mellitus, prenatal diagnosis, Z-score

## Abstract

Background: With increasing incidence of gestational diabetes mellitus (GDM), newborn infants with perinatal morbidity, including large-for-gestational-age (LGA) or macrosomia, are also increasing. The purpose of this study was to develop a prediction model for LGA infants with GDM mothers. Methods: This was a retrospective case-control study of 660 women with GDM and singleton pregnancies in four tertiary care hospitals from 2006 to 2013 in Korea. Biometric parameters were obtained at diagnoses of GDM and within two weeks before delivery. These biometric data were all transformed retrospectively into Z-scores calculated using a reference. Interval changes of values between the two periods were obtained. Multivariable logistic and stepwise backwards regression analyses were performed to develop the most parsimonious predictive model. The prediction model included pre-pregnancy body mass index (BMI), head circumference (HC), Z-score at 24 + 0 to 30 + 6 weeks’ gestation, and abdominal circumference (AC) Z-score at 34 + 0 to 41 + 6 weeks within 2 weeks before delivery. The developed model was then internally validated. Results: Our model’s predictive performance (area under the curve (AUC): 0.925) was higher than estimated fetal weight (EFW) within two weeks before delivery (AUC: 0.744) and the interval change of EFW Z-score between the two periods (AUC: 0.874). It was internally validated (AUC: 0.916). Conclusions: A clinical model was developed and internally validated to predict fetal overgrowth in Korean women with GDM, which showed a relatively good performance.

## 1. Introduction

Gestational diabetes mellitus (GDM) affects maternal, fetal, and neonatal well-being. The incidence of GDM has reached 14% in the United States of America [1]. It has increased from 2 to 13% worldwide [2,3]. GDM increases the risk of traumatic vaginal delivery, emergency cesarean section, postpartum hemorrhage, and large-for-gestational-age (LGA) or macrosomic infant delivery. LGA or macrosomic infants are at an increased risk of peripartum mortality due to complications such as shoulder dystocia, brachial plexus injury, bone fracture (clavicle or humerus), and birth asphyxia [4,5,6]. LGA newborns are also more likely to develop chronic health problems such as diabetes and metabolic diseases later in life [5,6,7]. Therefore, preventing fetal overgrowth through strict glycemic control and accurately predicting fetal overgrowth are crucial.

Several researchers have investigated specific equations to assess fetal growth using maternal factors and biomarkers [8,9] or to improve the predictive performance of fetal overgrowth among mothers with diabetes using biometric parameters such as abdominal circumference (AC), serial AC measurements, and estimated fetal weight (EFW) during the early third or third trimester [10,11,12,13].

Based on a comparison of weights among overweight newborns, EFW measurement at term is not as accurate as expected. Visualization of biometric parameters by ultrasonic imaging is limited due to distortion of the ultrasound beam when it penetrates deeper into the pregnant uterus of obese women [14]. Given inaccuracies caused by clinical characteristics of mothers, integration trials of clinical features to improve the prediction of LGA infant delivery are necessary.

Thus, the aim of this study was to develop a prediction model using clinical characteristics of pregnant women and fetal serial biometry based on Z-scores measured at GDM diagnosis and within two weeks before delivery to improve the detection of LGA fetus in mothers with GDM. An accurate model that could predict the risk of delivering an LGA baby would be useful for counseling women regarding risks of attempting a vaginal delivery and the timing of delivery in cases of suspected LGA infant delivery.

## 2. Materials and Methods

### 2.1. Study Design

This was a retrospective, case-control study of 660 women with GDM and singleton pregnancies at four tertiary care hospitals from 2006 to 2013 in Korea. We reviewed clinical information from medical records. Informed consent was not required due to the retrospective nature of this study. Fetal biometric parameters, such as biparietal diameter (BPD), head circumference (HC), AC, femur length (FL), and EFW measured during GDM diagnosis from 24 to 30 weeks of gestation (hereby indicated as BPD1, HC1, AC1, FL1, and EFW1), within 2 weeks before delivery at more than 34 weeks’ gestation (BPD2, HC2, AC2, FL2, and EFW2), and interval changes between the two periods in these values, were obtained from medical records. For example, interval changes of EFW and EFW Z-score between two periods are described as EFW/week [(EFW2 − EFW1)/(week2 − week1)] and Δ EFW Z-score.

BPD, HC, AC, and FL were converted to Z-scores and percentiles adjusted for gestational age according to reference charts and equations of Korean fetal biometry [15]. EFW was converted using INTERGROWTH-21st equation [16]. EFW as determined by ultrasonography was calculated using the Hadlock formula C [log10 EFW = 1.335 − 0.0034 (AC) (FL) + 0.0316 (BPD) + 0.0457 (AC) + 0.1623 (FL)] [17]. We then applied EFW (in grams) to the Alexander growth curve nomogram to calculate fetal weight percentage [18]. Based on the Alexander growth curve, an LGA fetus was suspected when the EFW by ultrasonography was >90%. An LGA infant at birth was defined as having a birth weight of ≥90%.

Exclusion criteria were: multiple gestations, major fetal anomalies and stillbirth, preterm birth of <34 weeks of gestation, and other medical and surgical problems except GDM. All ultrasonography examinations were routinely performed in each unit by skilled technicians or physicians. GDM was diagnosed using the Carpenter and Coustan criteria. Clinical and demographic data, including gestational age and birth weight, and specific details of neonatal outcomes were collected from medical records.

### 2.2. Data Analysis

Maternal demographic data were compared between LGA and appropriate-for-gestational-age (AGA) infants of mothers with GDM. Clinical variables were compared using Pearson’s *X*^2^ test, Student’s *t*-test, and the Mann–Whitney *U* test. Clinically significant factors, fetal biometric parameters, and their Z-scores were analyzed to evaluate a risk an LGA infant at birth by univariate logistic regression (Table 2). For a new prediction model, a backward stepwise variable elimination procedure was used to select the best goodness-of-fit model in multivariable binary logistic regression analysis. We performed a multiple binary logistic regression analysis to identify significant risk factors and to generate the most parsimonious prediction model. The developed model was internally validated by LOOCV (leave-one-out cross-validation). Significant predictors were converted to binary variables using receiver operator characteristic (ROC) curve analysis (response variables: LGA vs. AGA). For each predictive variable, we created a binary variable defined as “low” when it was smaller than the cutoff and “high” when it was larger than the cutoff. Significance was defined at *p* < 0.05. R language version 3.3.3 (R Foundation for Statistical Computing, Vienna, Austria), T&F program version 1.6 (YooJin BioSoft, Goyang, Korea), and IBM SPSS Statistics for Windows version 23 (IBM Corp., Armonk, NY, USA) were used for all statistical analyses.

## 3. Results

Among 660 women with GDM and singleton pregnancies, 77 (11.6%) had an LGA infant delivery. Maternal age and height were not significant factors for predicting LGA infants (Table 1). Delivery occurred at 38.67 ± 1.22 weeks and 38.1 ± 1.19 weeks of gestation in AGA and LGA groups, respectively (*p* < 0.001). The cesarean section rate in the LGA group was 75.3%, which was higher than that (48.2%) in the AGA group. Pre-pregnancy weight and BMI were significantly higher in women who delivered an LGA fetus compared to those who delivered an AGA fetus. LGA and AGA infants had mean birth weights of 3998 g and 3214 g, respectively. The significance level of the above-mentioned variables was *p* < 0.001.

Results of univariate binary logistic regression using type (LGA vs. AGA) are shown in Table 2. Among statistically significant results (*p* < 0.001), parameters with the highest odd ratios (OR) were AC1 Z-score (2.015; 95% confidence interval (CI): 1.612—2.518; *p* < 0.001) in gestational diagnosis periods, AC2 Z-score (6.15; 95% CI: 4.284—8.83; *p* < 0.001) in 2 weeks before delivery periods, and the interval change of AC between two periods (AC/week) (193.31; 95% CI: 44.516—839.503; *p* < 0.001). The Δ AC Z-score had the highest OR (1.889; 95% CI: 1.509—2.366; *p* < 0.001) among Z-scores between the two periods.

**Figure 1:**
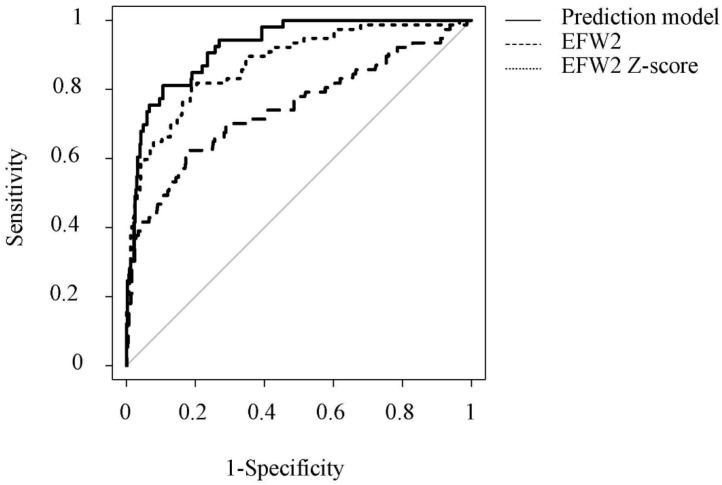
Comparison of ROC with EFW2 and EFW2 Z-score measured within two weeks before delivery and prediction model using type as binary response. ROC, receiver operator characteristic curve; EFW, estimated fetal weight.

In Table 3, the best predictive model consisted of three predictors (HC1 Z-score, AC2 Z-score, and pre-pregnancy BMI) by multivariable binary logistic regression model to predict LGA using dichotomous predictors. As shown in Table 4, AUCs for the ability of EFW2 alone and EFW2 Z-score to predict LGA infant delivery were 0.744 (95% CI: 0.675–0.813; *p* < 0.001) and 0.874 (95% CI: 0.874–0.919; *p* < 0.001), respectively. Meanwhile, the AUC of our prediction model was 0.925, with a sensitivity of 81.1% and a specificity of 89.5% (95% CI: 0.892–0.957; *p* < 0.001). In Table 4, the prediction model was internally validated by LOOCV (AUC: 0.916), which showed a sensitivity of 81.1% and a specificity of 87.4% (95% CI: 0.880–0.953; *p* < 0.001). Additionally, several diagnostic measures of the prediction performance of the internally validated prediction model (diagnostic OR: 29.84) were shown. Figure 1 presents results of comparison of ROC with EFW2, EFW2 Z-score, and prediction model using type as binary response.

**Figure 2:**
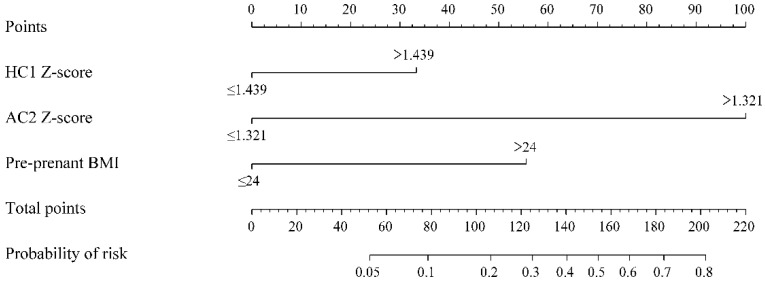
Nomogram for predicting an LGA infant in mothers with GDM. Directions: pre-pregnancy BMI, HC1 Z-score measured during GDM diagnosis, and AC2 Z-score measured within two weeks before delivery for an individual patient. Instructions: Each individual’s risk of delivering an LGA infant was estimated by plotting on each variable axis. The nomogram was able to predict LGA babies in pregnant women with GDM at the optimal cutoff values of HC1 Z-scores > 1.439, AC2 Z-scores > 1.321, and pre-pregnancy BMIs > 24, under which AGA babies could be considered. Points from each variable value were then summed. The sum of the total points scale was located and vertically projected onto the bottom axis. A probability risk for an LGA infant was then obtained. LGA, large for gestational age; GDM, gestational diabetes mellitus; BMI, body mass index; HC, head circumference; AC, abdominal circumference.

Based on univariate and multivariate binary analyses, the nomogram for predicting an LGA infant using cutoff of predictors is presented in Figure 2. The probability in the nomogram was determined using the following formula:

*P* = e^βX^/(1 + e^βX^)

where

βX = −4.469 + C1 ∗ HC1 Z-score + C2 ∗ AC2 Z-score + C3 ∗ Pre-pregnancy BMI;

C1 = {0, 0.965} when HC1 Z-score = {≤1.439, >1.439};

C2 = {0, 2.895} when AC2 Z-score = {≤1.321, >1.321};

C3 = {0, 1.609} when Pre-pregnancy BMI = {≤24, >24}.

## 4. Discussion

Our study has significant strengths. As mentioned above, we developed a prediction model by combining maternal pre-pregnancy BMI with biometric parameters adjusted for gestational age with Z-scores according to reference charts and equations from Korean reference data. The model with the highest diagnostic performance was selected and internally validated.

Another strength of our study was the creation of a clinically applicable nomogram. Using the nomogram, points for each category presented can be calculated and summed to assess the overall risk of LGA infant delivery in mothers with GDM. The total number of points of >0.8 and <0.05 corresponded to extremely high risk and extremely low risk, respectively. This will help improve the prediction of LGA infant delivery in women with GDM and allow clinicians to appropriately counsel women about potential risks. Moreover, compared with the predictive value of EFW or AC alone measured by ultrasonography during the third trimester, our prediction model had significantly higher predictive value for diagnosing LGA infant delivery. Although accurately estimating the fetal weight at delivery is often limited due to fetal malposition or maternal clinical status, appropriate counseling during pregnancy is possible using the created nomogram.

Previous researchers have proposed that growth acceleration of LGA infants begins in the second trimester and continues throughout the third trimester [19,20,21]. Considering this, we sought to create a model to predict LGA infants’ delivery in mothers with GDM.

To develop a predictive model, fetal biometric parameters such as BPD, AC, FL, and EFW measured at GDM diagnosis and within two weeks before delivery and interval changes between the two periods were obtained from medical records. The addition of clinical characteristics such as pre-pregnancy BMI significantly improved the prediction. Biometric parameters and clinical risk factors were combined through multivariable logistic analysis, carefully considering each factor and their possible interrelationships.

Predictors for LGA infant delivery in our model have been determined in prior studies. In several recent studies, ultrasonography-measured EFW in the third trimester was used to predict LGA infant delivery. In one study involving 1689 pregnant women who were within 8 days of delivery after 37 weeks of gestation, the detection rate at a 5% false-positive rate (FPR) for LGA infant delivery using EFW derived from fetal AC was 54% [22]. Canavan et al. showed that an AC of >90th percentile is the best predictor of macrosomia at birth when comparing EFW, AC, and FL measured during sonographic examinations at 28–34 weeks of gestation [23]. The prediction of LGA infant delivery using only EFW in the third trimester tends to be overestimated, and the detection rate tends to be inferior. In our study, the detection rate with EFW2 was inferior to that of our new model. AUCs computed using our prediction model and EFW2 were 0.925 (95% CI: 0.892–0.957) and 0.744 (95% CI: 0.675–0.813), respectively, which were significantly different (*p* < 0.001 using DeLong’s test).

Based on previous studies, attempts have been made to increase the prediction rate for LGA infant delivery by ultrasonographic findings in the third trimester as well as in the first or second trimester. Pilalis et al. reported that the detection rate for LGA infant delivery at >95th percentile is 31% at a 10% FPR using a combination of maternal weight and height, fetal crown–rump length, and delta nuchal translucency at 11–13 weeks of gestation [24]. The rate was increased to 52% after adding fetal biometry at 30–32 weeks. It was improved to 63% at 34–37 weeks in the same group [12,25]. Meanwhile, in a previous study, the ultrasonographic difference between fetal AD and BPD was used to predict macrosomia [26]. The improved accuracy of predicting LGA infant delivery could reduce the error between EFW and actual neonatal weight at delivery, thereby reducing complications that may occur during delivery and the rate of unnecessary cesarean sections.

This study had some limitations. First, GDM diagnosis in this paper is based on the Carpenter and Coustan criteria, whereas many countries are using IADPSG or WHO criteria. It should be considered that the LGA infant prediction with GDM mothers by our prediction model may perform differently used according to the IADPSG or WHO criteria. Second, the degree of glycemic control and treatment status were not included as a variable in the predictive model. This study considered HbA1C as a glycemic control variable. However, since it was conducted as a multicenter-retrospective study, the examination time of HbA1C test was different in each hospital, and many data were missing. Therefore, using it to develop a predictive model of LGA infants with HbA1C related to the degree of glycemic control had limitations. In addition, this study did not include variables between the group treated with oral hypoglycemic agents or insulin and without treatment and parameters related to adverse outcomes such as preeclampsia. Finally, the EFW Z-score calculation equation for our prediction model was applied using the INTERGROWTH 21 equation because it was not found among the Korean reference data. Considering that Asian data such as that from India, China, etc., were included compared to other studies, EFW was calculated by applying INTERGROWTH 21. We hope that as soon as the Korean reference data are developed, further study will be conducted based on them. A prospective study will be needed complementing for these limitations in the predictive model.

In conclusion, a clinical model was developed and internally validated to predict fetal overgrowth in Korean women with GDM, which showed a relatively good performance. By providing obstetricians and future mothers with an instrument in assessing fetal weight, it is expected to increase the predictive rate of LGA infants in GDM mothers. Applying clinically nomograms derived from this model will be helpful in reducing LGA births and improving a perinatal prognosis for mothers and newborns.

## Figures and Tables

**Table 1:** Demographic data of pregnant women with gestational diabetes mellitus based on type of infant delivery.

Variable	AGA (*n* = 583)	LGA (*n* = 77)	*p*-Value
Maternal age (years) *	33.74 ± 4.08	34.53 ± 4.29	0.139
Delivery (weeks) *	38.67 ± 1.22	38.1 ± 1.19	<0.001
Delivery mode ^‡^			<0.001
Vaginal delivery	302 (51.8)	19 (24.7)	
Cesarean section	281 (48.2)	58 (75.3)	
Height *	160.26 ± 5.51	160.92 ± 5.93	0.512
Pre-pregnancy weight *	58.53 ± 10.53	66.73 ± 12.37	<0.001
Pre-pregnancy BMI *	22.73 ± 3.82	25.81 ± 4.45	<0.001
Birth weight (g) ^†^	3214.32 ± 343.39	3998.77 ± 330.74	<0.001

Abbreviations: LGA, large for gestational age; AGA, appropriate for gestational age; BMI, body mass index. *^,†^: Values are given as mean ± standard deviation. ^‡^: Values are given as number (percentage). * Tested using the Mann–Whitney *U* test. ^†^ Tested using Student’s *t*-test. ^‡^ Tested using Pearson’s *χ*^2^ test.

**Table 2:** Result of univariable binary logistic regression analysis using type (LGA vs. AGA) according to gestational age.

Predictor	OR (95% CI)	*p*-Value
BPD1 Z-score	1.463 (1.16–1.844)	0.001
HC1 Z-score	1.449 (1.179–1.781)	<0.001 **
AC1 Z-score	2.015 (1.612–2.518)	<0.001 **
FL1 Z-score	1.086 (0.88–1.339)	0.441
EFW1 Z-score	1.935 (1.511–2.477)	<0.001 **
BPD2 Z-score	2.206 (1.643–2.962)	<0.001 **
HC2 Z-score	1.734 (1.263–2.382)	<0.001 **
AC2 Z-score	6.15 (4.284–8.83)	<0.001 **
EFW2 Z-score	8.438 (5.527–12.883)	<0.001 **
FL2 Z-score	1.91 (1.481–2.465)	<0.001 **
Pre-pregnancy BMI	1.169 (1.105–1.237)	<0.001 **
EFW2 (g)	1.002 (1.002–1.003)	<0.001 **
HC (cm)/Week	0.445 (0.07–2.847)	0.393
AC (cm)/Week	193.317 (44.516–839.503)	<0.001 **
EFW (g)/Week	1.041 (1.032–1.05)	<0.001 **
Δ HC Z-score	0.916 (0.737–1.139)	0.431
Δ AC Z-score	1.889 (1.509–2.366)	<0.001 **
Δ EFW Z-score	1.745 (1.383–2.202)	<0.001 **

**: *p* < 0.001. Abbreviations: LGA, large for gestational age; AGA, appropriate for gestational age; OR, odds ratio; 95% CI, 95% confidence interval of AUC; BMI, body mass index; BPD, biparietal diameter; HC, head circumference; AC, abdominal circumference; FL, femur length; EFW, estimated fetal weight; 1, fetal biometric parameters measured during screening of gestational diabetes mellitus; 2, fetal biometric parameters measured within 2 weeks before delivery. “Predictor/week” means predictor velocity. Predictor velocity between two scans described as predictor/week and calculated by [(predictor 2 − predictor 1)/(week2-week1)]. “Δ Predictor (Z-score)” means Z-score’s interval change of predictor between gestational weeks [(predictor 2 (Z-score) − predictor 1 (Z-score)].

**Table 3:** Results of multivariable logistic regression model to predict LGA using dichotomous predictors using type (LGA vs. AGA).

Predictor	Continuous Predictors		Binary Predictors		
	OR (95% CIs)	*p*-Value	OR (95% CIs)	*p*-Value	Cut-Off
HC1 Z-score	1.358 (1.055–1.748)	0.018	2.625 (1.23–5.601)	0.013	1.439
AC2 Z-score	6.345 (3.976–10.124)	<0.001	18.083 (8.35–39.164)	<0.001	1.321
Pre-pregnancy BMI	1.153 (1.055–1.26)	0.002	4.996 (2.405–10.378)	<0.001	24.02

Abbreviations: LGA, large for gestational age; AGA, appropriate for gestational age; OR, odds ratio; CI, confidence interval; BMI, body mass index; HC, head circumference; AC, abdominal circumference; 1, fetal biometric parameters measured during screening of gestational diabetes mellitus; 2, fetal biometric parameters measured within 2 weeks before delivery. The cutoff of predictor value was used for classifying binary response of type: control response, AGA vs. case response, LGA.

**Table 4:** Diagnostic performance of EFW2, estimated model, and validated model using type (LGA vs. AGA).

Predictor	AUC	Lo95% CI	Up95% CI	*p*-Value	Acc.	Sensi	Speci	FPR	FNR	PPV	NPV	LR+	LR−	DOR	Cut-Off
EFW2	0.744	0.675	0.813	<0.001	0.798	0.623	0.822	0.178	0.377	0.316	0.943	3.495	0.458	7.623	
EFW2 Z-score	0.874	0.829	0.919	<0.001	0.809	0.805	0.811	0.189	0.208	0.357	0.967	4.199	0.256	16.394	0.108
Estimated Model	0.925	0.892	0.957	<0.001	0.884	0.811	0.894	0.106	0.189	0.5	0.973	7.642	0.211	36.20	0.165
Validated Model	0.916	0.880	0.953	<0.001	0.867	0.811	0.874	0.126	0.189	0.457	0.973	6.443	0.216	29.84	

Abbreviations: EFW2, estimated fetal weight within 2 weeks before delivery; LGA, large for gestational age; AGA, appropriate for gestational age; AUC, area under the curve; 95% CI, 95% confidence interval; Sensi, sensitivity; Speci, specificity; Acc., accuracy; FPR, false-positive rate; FNR, false-negative rate; PPV, positive predictive value; NPV, negative predictive value; LR+, positive likelihood ratio; LR−, negative likelihood ratio; DOR, diagnostic odds ratio.

## Data Availability

Data presented in this study are available from the corresponding author upon reasonable request.
